# Diagnostic accuracy of neonatal structural MRI scores to predict 6-year motor outcomes of children born very preterm

**DOI:** 10.1016/j.nicl.2024.103725

**Published:** 2024-12-14

**Authors:** Karen H. Mistry, Samudragupta Bora, Kerstin Pannek, Alex M. Pagnozzi, Simona Fiori, Andrea Guzzetta, Robert S. Ware, Paul B. Colditz, Roslyn N. Boyd, Joanne M. George

**Affiliations:** a Child Health Research Centre, Faculty of Medicine, The University of Queensland, Brisbane, Australia; b Mater Research Institute, Faculty of Medicine, The University of Queensland, Brisbane, Australia; c Health Services Research Center, University Hospitals Research & Education Institute, Department of Pediatrics, University Hospitals Rainbow Babies & Children’s Hospital, Case Western Reserve University School of Medicine, Cleveland, USA; d The Australian e-Health Research Centre, CSIRO Health and Biosecurity, Brisbane, Australia; e School of Electrical Engineering and Computer Science, The University of Queensland, Brisbane, Australia; f Neuroscience and Medical Genetics Department, Meyer Children’s Hospital, Florence, Italy; g Department of Developmental Neuroscience, IRCCS Stella Maris Foundation, Pisa, Italy; h Department of Clinical and Experimental Medicine, University of Pisa, Pisa, Italy; i Griffith Biostatistics Unit, Griffith University, Brisbane, Australia; j The University of Queensland Centre for Clinical Research, The University of Queensland, Brisbane, Australia; k Perinatal Research Centre, Royal Brisbane and Women’s Hospital, Brisbane, Australia; l Queensland Cerebral Palsy and Rehabilitation Research Centre (QCPRRC), Child Health Research Centre, Faculty of Medicine, The University of Queensland, Brisbane, Australia; m Physiotherapy Department, Queensland Children’s Hospital, Children’s Health Queensland Hospital and Health Service, Brisbane, Australia

**Keywords:** Very preterm, Structural MRI, Motor outcome, Cerebral palsy, MABC-2, Diagnostic accuracy

## Abstract

**Aims:**

This study aimed to (1) evaluate associations between Early and Term structural MRI (sMRI) brain abnormality scores and adverse motor outcomes at 6-years corrected age (CA), (2) determine their diagnostic accuracy in predicting adverse motor outcomes and cerebral palsy (CP) at 6-years CA.

**Methods:**

Infants born < 31-weeks gestational age (GA) returning for 6-year follow-up were included. Early and Term sMRI were scored using a validated method, deriving white matter, cortical grey matter, deep grey matter, cerebellar and global brain abnormality scores (GBAS). At 6-years CA, Movement Assessment Battery for Children-2nd Edition (MABC-2) was administered. Linear regression assessed associations between Early and Term GBAS/subscale scores and 6-year MABC-2 total score. For diagnostic accuracy, sMRI scores were categorised as none/mild vs moderate/severe, MABC-2 cut-off ≤ 5th percentile, and CP as present/absent.

**Results:**

Infants had Early MRI (n = 123) at mean PMA 32.5-weeks (median GA 28.4-weeks; mean birthweight 1101 g) and n = 114 had Term MRI (Mean PMA 40.8-weeks). Nine had CP and n = 116 had MABC-2 scores. Early (B: −1.92; p ≤ 0.001) and Term (B: −1.67; p ≤ 0.01) GBAS were negatively associated with MABC-2 scores. Both Early and Term GBAS had high specificity (Sp) and low sensitivity (Se) in predicting MABC-2 ≤ 5th percentile (Early: Se 36 %, Sp 82 %; Term: Se 28 %, Sp 93 %) and predicted CP with high Se and Sp (Early: Se 78 %, Sp 78 %; Term: Se 75 %, Sp 89 %).

**Conclusion:**

High Sp of Early and Term MRI predicting an outcome on MABC-2 may help accurately identify infants unlikely to develop motor impairments at 6-years CA.

## Highlights

• Early and Term MRI brain abnormality scores had similar associations and predictive accuracy for 6-years motor outcomes.
• Structural MRI had stronger accuracy to predict cerebral palsy (CP) vs mild/moderate motor outcomes.
• Early and Term MRI showed high specificity, but low sensitivity for predicting MABC-2 outcome.
• Cerebellum scores on Early and Term MRI showed strongest associations to 6-year motor outcome.
• Early MRI deep grey matter score had strongest predictive accuracy for an outcome of CP.

## 1 Introduction

Globally an estimated 2 million infants are born very preterm (<32 weeks' gestational age; GA) each year (Ohuma et al., 2023). Despite advances in neonatal care, very preterm infants are still more likely to have adverse motor outcomes including Cerebral Palsy (CP) than full-term infants (Chung et al., 2020b, Evensen et al., 2020). While the overall prevalence of CP is declining, there is a growing prevalence of non-CP motor impairments such as Developmental Coordination Disorder (DCD) among very preterm infants (Chung et al., 2020a, Edwards et al., 2011, McGowan and Vohr, 2019, Spittle et al., 2021). Timely identification of poor motor outcomes allows for targeted early interventions to improve long-term outcomes.

While white matter injury (WMI) commonly occurs in the developing brain of preterm infants, injuries within the cortical grey matter (CGM), deep grey matter (DGM), and cerebellum have also been identified (Inder et al., 2023, Kidokoro et al., 2013). Following early brain injury, a secondary form of dysmaturation may affect preterm cerebral white and grey matter development, causing myelination disturbances, impaired synaptic development and subsequent volumetric changes (Back, 2015, Inder et al., 2023). Neonatal structural magnetic resonance imaging (sMRI) performed at term equivalent age (TEA) has been used to detect the presence of brain injury and resulting dysmaturation (Anderson et al., 2015). Kidokoro et al. (2013) developed a sMRI scoring system validated for use with TEA MRI (acquired between 36- and 42-weeks post menstrual age; PMA) to fully capture the impact of preterm birth, including brain injury and altered brain growth. The scoring system was associated with motor outcomes at 2-years (Kidokoro et al., 2014) and at 7-years corrected age (CA)(Anderson et al., 2017).

Emerging evidence suggests Early MRI (acquired < 36 weeks PMA) can predict later motor outcomes in preterm born children (George et al., 2018b). In light of this, the sMRI scoring system (Kidokoro et al., 2013) was validated for MRI acquired between 29–35 weeks PMA and found to be predictive of motor outcomes at 12-months CA and associated with outcomes at 2-years CA (George et al., 2021, George et al., 2017). There is currently no data on the diagnostic accuracy of this scoring method to predict motor outcomes later into childhood.

A greater understanding of the relationship of Early sMRI with later childhood outcomes could assist in using MRI to predict outcomes in a clinical setting. The aims of this study were to (1) evaluate the association between Early and TEA sMRI brain abnormality scores and adverse motor outcomes at 6-years CA, (2) determine the diagnostic accuracy of Early and TEA sMRI brain abnormality scores to predict adverse motor outcomes and CP at 6-years CA.

## 2 Materials and methods

### 2.1 Study Design and participants

Infants born < 31 weeks GA at Royal Brisbane and Women’s Hospital, Brisbane, Australia were recruited to prospective longitudinal cohort studies PPREMO, Prediction of PREterm Motor Outcomes (ANZCTR12613000280707); and PREBO, Prediction of Brain Outcomes, (ANZCTR12615000591550) between January 2013 to December 2018 (George et al., 2015, George et al., 2020). This cohort has been previously described (George et al., 2020). Infants with significant chromosomal or congenital abnormalities were excluded. Included infants had an Early MRI and returned for follow-up assessment at 6-years CA (PREBO-6 study; ANZTR12619000155190)(George et al., 2020). Ethics approval was granted by Children’s Health Queensland Hospital and Health Service Human Research Ethics Committee in February 2019 (HREC/19/QCHQ/49800) and The University of Queensland Human Ethics Research Committee in March 2019 (2019000426).

### 2.2 MRI acquisition

Infants had Early MRI at 30–32 weeks PMA or when medically stable, and repeat TEA MRI between 40–42 weeks PMA. Infants were scanned using a 3 T scanner Siemens Tim Trio (PPREMO) or Siemens Skyra (PREBO; Erlangen, Germany) in an incubator with MR compatible neonatal head coil (LMT Lammers Medical Technology, Lubeck, Germany)(George et al., 2015). Natus Mini Muffs (Natus Medical Inc., San Carlos, CA) were used to decrease noise from the scanner. Infants were scanned without sedation. Detail of MRI sequences used for PPREMO and PREBO cohorts have been previously described (Pagnozzi et al., 2023) and included in eMethods (Supplementary material).

### 2.3 MRI scoring

Scans were scored independently by a neurologist with training in radiology and experience in neonatal MRI scoring (S.F.) blinded to all clinical history except PMA at MRI. The Kidokoro et al. (2013) scoring method which was modified and previously described (George et al., 2017), was used to derive brain abnormality subscale scores accounting for presence of cystic lesions, signal abnormalities and volume reductions. These produced scores for cerebral white matter (WM; score 0–15), CGM (score 0–8), DGM (score 0–6) and cerebellum (score 0–6). A global brain abnormality score (GBAS) was derived from the sum of WM, CGM, DGM, and cerebellar values (score 0–35) with higher scores indicating more severe brain abnormality (George et al., 2017). The WM subscale scores were further categorized as none (0–2), mild (3–4), moderate (5–6) or severe (≥7). The CGM, DGM and cerebellum subscale scores were similarly categorized as none (0), mild (1), moderate (2) or severe (≥3). A total GBAS score between 0 and 3 was classified as normal, 4–7 as mild, 8–11 as moderate and a score ≥ 12 as severe brain abnormality (George et al., 2017). The raw regional measures for each subscale score were adjusted for PMA at MRI separately for each cohort, and age-adjusted regional measures of the PREBO cohort were additionally adjusted to match age-adjusted regional measures of the PPREMO cohort (Pannek et al., 2024). Subscale and GBAS continuous measures were converted to categories of none, mild, moderate and severe brain abnormality (George et al., 2017, Kidokoro et al., 2013).

### 2.4 Motor outcome at 6-years CA

At 6-years CA motor outcomes were assessed by experienced physiotherapists blinded to MRI findings and medical history, using Movement Assessment Battery for Children second edition (MABC-2) (Alison et al., 2018, Evensen et al., 2020). The MABC-2 evaluates manual dexterity, balance, and aiming and catching with eight motor item tasks. Raw scores were transformed into item standard scores, which combined to give the total test score (maximum score of 152), with lower scores indicating poorer motor performance. Total test scores were converted to percentile ranks, with ≤ 5th percentile indicating significant motor difficulties and 6th–15th percentiles indicating a child at risk of movement difficulties (Henderson et al., 2007). Cerebral Palsy was defined as a confirmed diagnosis made by the child’s pediatrician with severity documented using Gross Motor Function Classification System (GMFCS)‍.

### 2.5 Statistical analysis

Descriptive statistics for demographic and clinical characteristics are presented as median [interquartile range] or mean (standard deviation) for continuous data and frequency (percentage) for categorical data. Normality of continuous data was assessed using visual inspection of histograms and confirmed using the Shapiro–Wilk test, with P-value > 0.05 indicating a normal distribution. Continuous data were compared using Student’s *t*-test and the Mann-Whitney *U* test, depending on data distribution, while Pearson's X^2^ test was used for categorical data. Associations between sMRI brain abnormality scores, and motor outcome at 6-years CA were sought using univariable and multivariable linear regression models. Multivariable models included GA at birth, sex and CA at assessment as covariables. The associations were evaluated for Early and TEA sMRI subscale scores and GBAS separately, using MABC-2 total score as a continuous variable. Diagnostic accuracy was calculated using two by two tables, with a sensitivity (Se) and specificity (Sp) of ≥ 75 % considered high and ≤ 74 % as low. Subscale scores and GBAS on sMRI were dichotomized as normal/mild or moderate/severe classification categories. Cerebral palsy was dichotomized as the presence/absence, and MABC-2 percentile scores were dichotomized at 5th percentile. Additional sensitivity analysis was carried out using MABC-2 score dichotomized at 15th percentile. Receiver operating characteristic (ROC) curves were used to determine sMRI subscale and GBAS cut-off scores that maximised Se and Sp for motor outcomes. Statistical significance was defined as P < 0.05. Analyses were performed using Stata statistical software, v17 (StataCorp, College Station, TX).

## 3 Results

### 3.1 Clinical characteristics

Of 194 eligible preterm infants from PPREMO and PREBO studies, 123 returned for follow-up at 6-years CA in the PREBO-6 study. Demographic data is presented in Table 1. There were no significant differences in neonatal characteristics between infants who did, and did not return for follow-up (Table 1). Infants included in analyses had a median GA of 28.4 weeks, mean birthweight 1101 g and 57 % were male. All had an Early MRI (mean PMA 32.5 weeks), and n = 114 had a repeat MRI at mean PMA 40.8 weeks. The MRI scores and 6-year CA motor outcomes are summarised in Table 2 and Table 3 respectively. Proportions of infants across various severity categories (none, mild, moderate and severe) on Early and TEA for each subscale score and GBAS are presented in eTable 1. At 6.1-years CA, n = 116 children completed the MABC-2 (mean total score 63). Three children with CP did not attempt an MABC-2 assessment as it was not suitable, while six completed the assessment and their results were included in the analysis. Four children without a CP diagnosis partially completed the MABC-2 but did not have total or percentile scores for analysis.

**Table 1:** Characteristics of preterm children who were followed up vs not followed up at 6-years corrected age.

Characteristics	Followed up at 6 years CA (*n* = 123)	Not Followed up (*n* = 71)	*P-* Value
Gestation age at birth (week)	28.4 [26.7–29.9]	29.0 [27.6–29.6]	0.20
Birth weight (g)	1100.5 (317.8), 524–1764	1149.2 (309.9), 494–1886	0.15
Birth head circumference (cm)	25.8 (2.3), 20.2–30.5, *n =* 120	26.3 (2.2), 20.5–30.5,*n =* 68	0.08
Males	70 (57 %)	41 (58 %)	0.91
Multiple births	38 (31 %)	23 (32 %)	0.07
Premature rupture of membranes	31 (25 %)	12 (17 %)	0.18
Caesarean section	88 (72 %)	48 (68 %)	0.56
Antenatal steroids	90 (73 %)	47 (66 %)	0.30
Chorioamnionitis	23 (19 %), *n =* 122	9 (13 %)	0.27
Magnesium sulphate	65 (53 %), *n =* 122	41 (58 %)	0.35
High social risk^α^	60 (49 %), *n =* 122	39 (56 %), *n =* 70	0.38

Acquired medical factors			
Patent ductus arteriosus	50 (41 %)	25 (35 %)	0.45
IVH	40 (37 %), *n =* 109	16 (25 %), *n =* 64	0.11
IVH grade 3 or 4	14 (13 %), *n =* 109	6 (9 %), *n =* 64	0.60
Periventricular leukomalacia	5 (4 %)	1 (1 %)	0.30
Hydrocephalus	7 (6 %)	2 (3 %)	0.36
NEC diagnosed or suspected	4 (3 %)	5 (7 %)	0.23
Confirmed sepsis	4 (3 %)	4 (6 %)	0.42
Total parenteral nutrition (days)	11 [9–14]	12 [7–15], *n =* 69	0.61
Postnatal corticosteroids	21 (17 %)	7 (10 %)	0.17
Ventilation (days)	3 [2–20], *n =* 119	3 [1–10], *n =* 69	0.33
CPAP (days)	25 [7–44]	28 [8–46]	0.95
Oxygen therapy (hours)	79 [5–882], *n =* 121	78 [5–665]	0.93
Bronchopulmonary dysplasia	46 (38 %), *n =* 120	24 (35 %), *n =* 69	0.63
PMA at Early MRI, weeks	32.5 (1.4), 29.4–35.3	32.1 (1.2), 29.6–35.3	0.94
PMA at TEA MRI, weeks	40.8 (1.2), 38.7–46.6, *n =* 114	40.8 (1.0), 38.4–44.0, *n =* 59	0.59

Key: Data presented as Number (%), median [IQR] or mean (SD), range; CA, corrected age; IVH, intraventricular haemorrhage; NEC, necrotizing enterocolitis; CPAP, continuous positive airway pressure; PMA, post menstrual age; MRI, magnetic resonance imaging; TEA, term equivalent age; ^α^Social risk is assessed using a questionnaire that examines six areas, each scoring 0–2 (max score 12), including family structure, maternal age, education level, occupation, employment status, and language spoken at home. Scores of ≥ 2 were classified as indicating higher social risk.

**Table 2:** Brain MRI scores.

MRI Scores	Early MRI (n = 123)	TEA MRI (n = 114)	P-values
PMA at MRI [mean weeks (SD)]	32.5 (1.4)	40.8 (1.2)	
White matter	4 [3–5], 2–12	2 [1–4], 0–11	<0.001*
Cortical grey matter	0 [0–1], 0–2	0 [0–1], 0–3	0.03*
Deep gray matter	0 [0–1], 0–6	0 [0–1], 0–6	<0.001*
Cerebellum	0 [0–1], 0–5	0 [0–1], 0–5	<0.001*
GBAS	5 [3–8], 2–22	3 [1–5], 0–20	<0.001*

Key: Data presented as median [IQR], range; Early MRI white matter score range: 2–15 as all infants given a score of 2 for myelination delay as it was not expected by 36 weeks PMA; TEA MRI white matter score range 0–15; cortical grey matter score range 0–8; deep grey matter score range 0–6, cerebellum score range 0–6; global score range: Early 2–35 and TEA 0–35; GBAS, global brain abnormality score.

**Table 3:** Motor outcomes at 6-years corrected age.

Assessment	Scores (n = 123)
Corrected age at assessment (years)	6.1 (0.1), 6.0–6.8
MABC-2 total test score	63.3 (20.0), 12–108, *n =* 116
MABC-2 percentile score (%)	28.2 (29.1), 0.1–99.9, *n =* 116
MABC-2 ≤ 5th percentile	39 (34 %), *n =* 116
MABC-2 6th to 15th percentile	10 (8 %), *n =* 116
Cerebral Palsy	9 (7 %)
GMFCS level I	4 (3 %)
GMFCS level II	3 (2 %)
GMFCS level IV	2 (1 %)

Key: Data presented as mean (SD), range, and number (%); MABC-2, movement assessment battery for children second edition; GMFCS, gross motor function classification scale.

### 3.2 Association between MRI and MABC-2 motor outcome

**Fig. 1:**
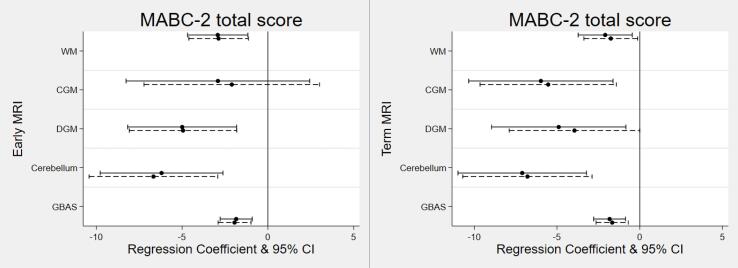
**Association between MRI and MABC-2 motor outcome;Description:** Association between Early (left) and Term (right) MRI scores and Movement Assessment Battery for Children, second edition (MABC-2) total test score at 6 years corrected age. Solid lines represent univariable regression analyses and dashed lines represent multivariable analyses adjusted for gestational age, sex and corrected age at motor assessment.

Univariable and multivariable regression analyses are presented in Fig. 1 (eTable 2 in Supplement). Higher GBAS on both Early (B: −1.92; 95 % confidence interval (CI): −2.88, −0.96) and TEA MRI (B: −1.67; 95 % CI: −2.64, −0.69) were associated with poorer motor outcomes as demonstrated by a lower total MABC-2 test score at 6-years CA (eFigure 1). The cerebellum subscale score had the strongest association with MABC-2 total test score at both Early (B: −6.67; 95 % CI: −10.42, −2.92) and TEA MRI (B: −6.79; 95 % CI: −10.70, −2.88).

### 3.3 Predicting motor outcomes on MABC-2 at 6-years

Diagnostic accuracy of Early and TEA MRI scans to predict MABC-2 scores ≤ 5th percentile, are presented in Table 4. Results of ROC analyses determined an MRI score dichotomized as none/mild and moderate/severe provided the best combination of Se and Sp for most subscale MRI scores and GBAS on Early and TEA MRI predicting MABC-2 outcomes (Supplement; eTable 4, eTable 5, eFigure 2, eFigure 3). Early MRI GBAS of moderate/severe brain abnormality had the strongest predicative accuracy for MABC-2 ≤ 5th percentile with a high Sp of 82 % (95 % CI: 75, 88) but low Se of 36 % (95 % CI: 27, 45). Similarly, TEA MRI moderate/severe GBAS had a high Sp of 93 % (95 % CI: 88, 98) and low Se of 28 % (95 % CI: 19, 36), but correctly classified a higher proportion of children (71 %) than other subscale scores. Additional sensitivity analysis on Early and Term MRI subscale scores and GBAS demonstrated on average 5 % more children were correctly classified with an outcome ≤ 5th percentile compared to ≤ 15th percentile on results from two by two tables (eTable 3) and ROC analysis (eTable 4).

**Table 4:** Sensitivity and specificity of Early and TEA MRI scores to predict MABC-2 ≤ 5th percentile and CP at 6-years corrected age. (results from 2x2 tables).

Variables	Sensitivity [95 % CI]	Specificity [95 % CI]	Positive likelihood ratio [95 % CI]	Negative likelihood ratio [95 % CI]	Correctly classified (%)
Cerebral Palsy					
Early MRI, *n* = 123					
WM	78 [70, 85]	74 [66, 81]	2.96 [1.86, 4.71]	0.30 [0.09, 1.03]	74
CGM	11 [6, 17]	89 [83, 94]	0.97 [0.14, 6.63]	1.00 [0.79, 1.28]	83
DGM	78 [70, 85]	89 [83, 94]	6.82 [3.67, 12.67]	0.25 [0.07, 0.85]	88
Cerebellum	33 [25, 42]	88 [82, 94]	2.71 [0.95, 7.73]	0.76 [0.48, 1.21]	84
GBAS	78 [70, 85]	78 [71, 85]	3.55 [2.17, 5.80]	0.28 [0.08, 0.97]	88
TEA MRI, *n* = 114					
WM	75 [67, 83]	86 [79, 92]	5.30 [2.86, 9.82]	0.29 [0.09, 0.97]	85
CGM	38 [29, 46]	80 [73, 87]	1.89 [0.72, 5.01]	0.78 [0.45, 1.34]	77
DGM	25 [17, 33]	86 [79, 92]	1.77 [0.49, 6.41]	0.87 [0.58, 1.31]	82
Cerebellum	50 [41, 59]	92 [86, 97]	5.89 [2.32, 14.97]	0.55 [0.27, 1.10]	89
GBAS	75 [67, 83]	89 [83, 95]	6.63 [3.40, 12.90]	0.28 [0.08, 0.94]	67

MABC-2 ≤ 5th percentile cut-off score					
Early MRI, *n* = 116					
WM	38 [30, 47]	77 [69, 84]	1.65 [0.92, 2.90]	0.80 [0.61, 1.06]	64
CGM	13 [7, 19]	90 [84, 95]	1.23 [0.43, 3.52]	0.97 [0.84, 1.12]	64
DGM	26 [18, 34]	91 [86, 96]	2.82 [1.16, 6.84]	0.82 [0.67, 1.00]	69
Cerebellum	23 [15, 31]	90 [84, 95]	2.22 [0.93, 5.31]	0.86 [0.71, 1.04]	67
GBAS	36 [27, 45]	82 [75, 88]	1.97 [1.05, 3.72]	0.78 [0.61, 1.01]	66

TEA MRI, *n* = 108					
WM	31 [22, 39]	90 [85, 96]	3.14 [1.33, 7.42]	0.77 [0.61, 0.97]	70
CGM	25 [17, 33]	85 [78, 92]	1.64 [0.75, 1.09]	0.89 [0.72, 1.09]	65
DGM	17 [10, 24]	88 [81, 94]	1.33 [0.51, 3.46]	0.95 [0.80, 1.13]	64
Cerebellum	19 [12, 27]	93 [88, 98]	2.80 [0.95, 8.21]	0.87 [0.73, 1.03]	69
GBAS	28 [19, 36]	93 [88, 98]	4.00 [1.48, 10.83]	0.78 [0.63, 0.96]	71

Key: Presented data in %, and % [95 % confidence interval (CI)]; WM, white matter; CGM, cortical grey matter; DGM, deep grey matter; GBAS, global brain abnormality score.

### 3.4 Predicting outcomes of Cerebral Palsy at 6-years CA

The ROC curves determined cut-off MRI GBAS of moderate/severe to derive the highest Se and Sp for predicting CP on Early and TEA MRI. Despite comparable Se and Sp values when using GBAS as categorical or continuous measures on ROC analysis, using continuous scores yielded higher correctly classified children (92 % on Early and TEA MRI) compared to categorical scores (78 % Early MRI; 88 % TEA MRI) (Supplement; eTable 6, eFigure 2, eFigure 3). Early MRI GBAS moderate/severe had a high Se of 78 % (95 % CI: 70, 85) and Sp of 78 % (95 % CI: 71, 85) for predicting CP at 6-years CA (Table 4). Early MRI DGM moderate/severe score had the strongest predictive accuracy for CP at 6-years CA (Se 78 %, 95 % CI: 70, 85; Sp 89 %, 95 % CI: 83, 94). Term MRI GBAS moderate/severe score had a Se of 75 % (95 % CI: 67, 83) and Sp of 89 % (95 % CI: 83, 95) for predicting CP. Despite having similar Se and Sp for predicting CP as GBAS, WM moderate/severe scores on TEA MRI (Se 75 %, 95 % CI: 67, 83; Sp 86 %, 95 % CI: 79, 92) correctly classified 85 % of children, whereas GBAS correctly classified 67 % (Table 4).

## 4 Discussion

Early and TEA MRI GBAS were associated with MABC-2 total test score at 6-years CA in this cohort of preterm born infants. The cerebellum score on both Early and TEA MRI had the strongest association with MABC-2 total test scores. Early MRI had higher Sp than Se in predicting an adverse motor outcome on MABC-2 percentile score, also observed at TEA MRI. Early and TEA MRI GBAS had high Se and Sp for predicting an outcome of CP at 6-years CA.

When examining the most common types of brain injuries in preterm infants (Inder et al., 2023), white matter injury (Cayam-Rand et al., 2019, Cayam-Rand et al., 2021), intraventricular haemorrhage (Benavente-Fernandez et al., 2019, Christensen et al., 2023), and cerebellar hemorrhage (Garfinkle et al., 2020) on Early MRI, were associated with poor motor outcomes on MABC-2 (≤5th and ≤ 15th percentile) between 4.5 and 8-years. When examining the subsequent impaired brain growth consequences of brain injuries among preterm infants referred to as brain dysmaturation (Inder et al., 2023), lower thalamus volume was associated with adverse motor outcomes on MABC-2 at 6 (Duerden et al., 2020) and 8-years of age (Cayam-Rand et al., 2021). Earlier publications on the same cohort as the current study, using similar sMRI scoring method assessing both brain injury and brain growth impairment found an association with adverse motor outcomes at 12- and 24-months CA (George et al., 2021, George et al., 2017). Consistent with previous findings on this cohort, the current study found Early and TEA MRI subscale scores and GBAS were negatively associated with MABC-2 total score at 6-years CA. The consistently observed association between sMRI scores and motor outcomes across different ages highlights there may be clinical utility potential for this scoring method.

In a prior study involving the same cohort, Early MRI cerebellar subscale score had the strongest association with concurrent clinical assessments of neurological and motor function in the neonatal period (George et al., 2018a). The association persisted to 6-years, since the present study indicated that the cerebellar score on Early and TEA MRI had the strongest association with motor outcomes at 6-years CA, highlighting the importance of the cerebellum in the development of motor skills among preterm infants (Stoodley and Limperopoulos, 2016). In this study, CGM showed no association with the MABC-2 total score on Early MRI but demonstrated the second strongest association after the cerebellum on TEA MRI. While these findings may reflect the biological processes of injury, they should be interpreted with caution due to the CGM subscale's lower interrater reliability compared to other MRI subscales reported during validation (George et al., 2017). A large number of infants in this study were noted to have mild brain abnormality on the WMI subscale score on Early MRI (55 %; score between 3–4 out of 15) as compared to no injury (15 %; score between 0–2 out of 15); however, on repeat TEA MRI there was a larger portion with no injury (62 %) as compared to mild injury (19 %). While rapid brain growth may explain the difference in WMI subscale scores between the two time-points, it should be noted that all infants were given a score of 2 for myelination delay on Early MRI because it was not expected by 36 weeks PMA, unlike on TEA MRI.

A systematic review and *meta*-analysis found Early MRI GBAS classified as normal/mild versus moderate/severe brain abnormality had a higher Sp (98 %) than Se (89 %) for predicting an adverse outcome at 2-years CA, on Bayley Scales of Infant Development, Second Edition below 2 standard deviations (George et al., 2018b). Early MRI GBAS in the present study demonstrated a consistent pattern of high Sp (82 %) and low Se (35 %) for predicting an adverse motor outcome on MABC-2 at ≤ 5th percentile at 6-years CA. Compared to 2-years (George et al., 2018b), Se at 6-years in our study was considerably lower, but it should be noted that the two studies included in the *meta*-analysis had very small samples of n = 12 (Drobyshevsky et al., 2007) and n = 31 (Sie et al., 2005) participants, respectively. Previous large prospective sample studies examining individual brain injuries using Early MRI found a similar pattern of low Se and high Sp for severe WMI (Se 21 %, Sp 89 %; n = 156) and cerebellar haemorrhage (Se 28 %, Sp 88 %; n = 165), to predict an outcome on MABC-2 after 4.5 years that was closer to the Se observed in this study (Christensen et al., 2023, Garfinkle et al., 2020). Additionally, brain abnormality on TEA MRI also had a lower Se (63 %) and higher Sp (73 %) for predicting an adverse motor outcome at ≥ 18 months CA (Van’t Hooft et al., 2015). The current study found TEA MRI global classified score predicted an MABC-2 score of ≤ 5th percentile, with a Se of 23 % and Sp of 93 %, similar to our findings for Early MRI. Notably, participant characteristics such as the intensity and duration of medical treatment, high social risk or interventions received prior to 6-year follow-up, may have contributed to the observed lower Se of MRI scores in predicting adverse outcomes on the MABC-2. While the influence of these factors on diagnostic accuracy was not examined in this study, it may warrant further investigation in future research. Our findings indicate that neonatal sMRI abnormality scores with lower Se may miss children who develop an adverse motor outcome at 6-years of age. Nevertheless, both Early and TEA MRI with high Sp could accurately identify infants without an adverse motor impairment at 6-years, with a reduced likelihood of false positives.

In line with previous literature both Early and TEA MRI GBAS had a higher Se and Sp for predicting an outcome of CP compared to overall adverse motor outcomes (George et al., 2018b, van’t Hooft et al., 2015). Early MRI GBAS predictive accuracy for detecting CP at 6-years in this study (Se 78 %, Sp 78 %), was lower than the published Se of 100 % and Sp of 93 % for predicting CP at 2-years (George et al., 2018b). Notably, the studies included in the *meta*-analysis had low sample sizes (n = 12(Drobyshevsky et al., 2007) n = 25(Atkinson et al., 2008) and n = 31(Sie et al., 2005)) and a prevalence of CP between 13 % and 28 %, compared to 7 % in this study. Our cohort also included more children with mild CP with GMFCS I and II than severe CP. These factors may have contributed to our study's lower predictive accuracy when compared to previous research.

Structural MRI subscale scores and GBAS had a higher Se for predicting CP, but a relatively lower Se for predicting mild to moderate adverse motor outcome on MABC-2. This highlights sMRI to have a stronger predictive accuracy for detecting severe outcomes as compared to predicting milder outcomes. To close this gap, future research could combine MRI findings with clinical data like perinatal characteristics to improve sensitivity for mild to moderate outcomes. Given the low Se of sMRI for predicting adverse motor outcome on MABC-2 in the current study, one could contend the utility of such a costly diagnostic test among very preterm infants. Despite this, parents of preterm infants seek balanced information, desiring to understand both the positives and negatives (Bell and Rysavy, 2018, Janvier et al., 2016). For families, the findings of high specificity for predicting adverse motor outcomes is valuable as it can provide reassurance by helping identify children who are less likely to have an adverse motor outcome. Additionally, the discussion regarding the usefulness of MRI scans for very preterm infants persists (Inder et al., 2021, Smyser et al., 2012). The MRI scans however, are recognised to be safe in this population, providing improved ability to evaluate brain injury and development, as well as valuable prognostic information to predict long term neurodevelopmental outcomes (Inder et al., 2023).

An important consideration for timing of neonatal MRI scan is that preterm infants may be discharged from the hospital prior to reaching TEA, necessitating a return trip to hospital for MRI. This could pose a risk of loss to follow-up, while incurring additional costs for the hospital to book an appointment post discharge. Identifying infants at risk of adverse motor outcomes prior to discharge from the neonatal intensive care unit (NICU) may support planning appropriate follow-up services. Furthermore, motor impairments in very preterm born children could co-occur with cognitive and behavioural difficulties (Foulder-Hughes and Cooke, 2003), affecting their overall quality of life, thus, emphasizing the importance of detecting and addressing the long-term effects of preterm birth. Obtaining an Early MRI before being discharged from the hospital could therefore provide substantial benefits for infants, their families, and the healthcare system.

Our study had several notable strengths. First, the study included infants who underwent an Early MRI < 36 weeks PMA, and the majority of these infants had a repeat scan at TEA. By conducting both Early and TEA MRI scans on the same cohort, we were able to perform analysis to evaluate their association with later motor outcome, while also comparing the results obtained at each scan time-point. Second, we reported both associations and diagnostic accuracy results to highlight accuracy of Early and TEA MRI to predict adverse motor outcomes at 6-years CA. Limitations of the present study include a 63 % follow-up rate, which was influenced by participants turning 6-years CA during the global COVID-19 pandemic. This resulted in some families being unable to visit our research facility due to interstate travel restrictions, immunisation status, or other medical reasons. Despite this limitation, the participants lost to follow-up had similar neonatal clinical characteristics as the infants followed up at 6-years, increasing confidence that the lower retention rate has not biased the analyses. A potential limitation of this study is that the two prospective cohorts were scanned on different scanners using different image acquisition protocols. It should be emphasised that we performed data harmonisation to reduce its effects(Pannek et al., 2024) and additional sensitivity analysis revealed no significant association between the included cohorts and motor outcomes. Furthermore, the neonatal MRI was scored by a single neurologist, which limited our ability to test inter-rater reliability for this scoring method. Future research could investigate how the combination of Early and TEA sMRI scores, and the combination of MRI, clinical characteristics and clinical assessment findings predict adverse motor outcomes later in childhood.

## 5 Conclusions

Both Early and TEA sMRI GBAS were negatively associated with adverse motor outcomes, with high Sp and low Se for predicting adverse motor outcomes at 6-years CA (≤5th percentile MABC-2). Early and TEA MRI had high Se and Sp for predicting CP at 6-years. Given the comparable findings in this study between Early and TEA MRI, sMRI acquired prior to term could be used to predict adverse motor outcomes later in childhood and inform discharge planning for this high-risk population earlier in the neonatal period. With growing prevalence of non-CP motor impairments, predicting these adverse outcomes remains challenging and warrants further research.

## Funding support

The study was supported by Cerebral Palsy Alliance Research Foundation [IRG1413: PBC, RNB, JMG], Financial Markets Foundation for Children [2014–074: PBC, RNB, JMG], National Health and Medical Research Council [NHMRC1084032: KP, RSW, PBC, RNB, JMG; NHMRC1161998: SB, KP, AMP, RSW, PBC, RNB, JMG], and Clinical Centre of Research Excellence (Australian Cerebral Palsy Clinical Trials Network) [NHMRC1116442: RB, JMG]. Prof Roslyn Boyd received an Investigator Fellowship grant from National Health and Medical Research Council [NHMRC1195602]. Dr Joanne George was supported by Queensland Government Health Practitioner Stimulus Grant [AH000762] and Mary McConnel Career Boost Program for Women in Paediatric Research [WIS162020]. Dr Samudragupta Bora was supported by a Mater Foundation Principal Research Fellowship and the University Hospitals Cleveland Medical Center and Case Western Reserve University School of Medicine's Joint Strategic Research Investment. Prof Andrea Guzzetta was supported by the Italian Ministry of Health (Linea 1 RC-2024 and 5 X 1000 Health Research voluntary contributions). Karen Mistry was supported by The University of Queensland Research Training Program Stipend Earmarked and Research Training Program Tuition Fee Offset.

## CRediT authorship contribution statement

**Karen H. Mistry:** Writing – original draft, Visualization, Methodology, Investigation, Formal analysis, Data curation. **Samudragupta Bora:** Writing – review & editing, Supervision, Resources, Project administration, Methodology, Funding acquisition, Conceptualization. **Kerstin Pannek:** Writing – review & editing, Supervision, Methodology, Investigation, Funding acquisition, Formal analysis, Data curation, Conceptualization. **Alex M. Pagnozzi:** Writing – review & editing, Supervision, Funding acquisition, Formal analysis, Conceptualization. **Simona Fiori:** Writing – review & editing, Methodology, Data curation. **Andrea Guzzetta:** Writing – review & editing, Methodology, Conceptualization. **Robert S. Ware:** Writing – review & editing, Supervision, Methodology, Funding acquisition, Formal analysis, Data curation, Conceptualization. **Paul B. Colditz:** Writing – review & editing, Supervision, Resources, Methodology, Investigation, Funding acquisition, Data curation, Conceptualization. **Roslyn N. Boyd:** Writing – review & editing, Supervision, Resources, Methodology, Funding acquisition, Data curation, Conceptualization. **Joanne M. George:** Writing – review & editing, Supervision, Resources, Project administration, Methodology, Investigation, Funding acquisition, Data curation, Conceptualization.

## Declaration of Competing Interest

The authors declare that they have no known competing financial interests or personal relationships that could have appeared to influence the work reported in this paper.

## Data Availability

Data will be made available on request.

## References

[b0005] Alison G., Rachel T., Prue E.M. (2018). Psychometric properties of gross motor assessment tools for children: a systematic review. BMJ Open.

[b0010] Anderson P.J., Cheong J.L.Y., Thompson D.K. (2015). The predictive validity of neonatal MRI for neurodevelopmental outcome in very preterm children. Semin. Perinatol..

[b0015] Anderson P.J., Treyvaud K., Neil J.J. (2017). Associations of newborn brain magnetic resonance imaging with long-term neurodevelopmental impairments in very preterm children. J. Pediatr..

[b0020] Atkinson J., Braddick O., Anker S. (2008). Cortical vision, MRI and developmental outcome in preterm infants. Arch Dis Child Fetal Neonatal Ed.

[b0025] Back S.A. (2015). Brain injury in the preterm infant: new horizons for pathogenesis and prevention. Pediatr. Neurol..

[b0030] Bell E.F., Rysavy M.A. (2018). What parents want to know after preterm birth. J. Pediatr..

[b0035] Benavente-Fernandez I., Synnes A., Grunau R.E. (2019). Association of socioeconomic status and brain injury with neurodevelopmental outcomes of very preterm children. JAMA Netw. Open.

[b0040] Cayam-Rand D., Guo T., Grunau R.E. (2019). Predicting developmental outcomes in preterm infants: a simple white matter injury imaging rule. Neurology.

[b0045] Cayam-Rand D., Guo T., Synnes A. (2021). Interaction between preterm white matter injury and childhood thalamic growth. Ann. Neurol..

[b0050] Christensen R., Chau V., Synnes A. (2023). Preterm neurodevelopmental trajectories from 18 months to 4.5 years. J. Pediatr..

[b0055] Chung E.H., Chou J., Brown K.A. (2020). Neurodevelopmental outcomes of preterm infants: a recent literature review. Translational Pediatrics..

[b0060] Chung E.H., Chou J., Brown K.A. (2020). Neurodevelopmental outcomes of preterm infants: a recent literature review. Translational Pediatrics..

[b0065] Drobyshevsky A., Bregman J., Storey P. (2007). Serial diffusion tensor imaging detects white matter changes that correlate with motor outcome in premature infants. Dev. Neurosci..

[b0070] Duerden E.G., Grunau R.E., Chau V. (2020). Association of early skin breaks and neonatal thalamic maturation: a modifiable risk?. Neurology.

[b0075] Edwards J., Berube M., Erlandson K. (2011). Developmental coordination disorder in school-aged children born very preterm and/or at very low birth weight: a systematic review. J. Dev. Behav. Pediatr..

[b0080] Evensen K.A.I., Ustad T., Tikanmäki M. (2020). Long-term motor outcomes of very preterm and/or very low birth weight individuals without cerebral palsy: a review of the current evidence. Semin. Fetal Neonatal Med..

[b0085] Foulder-Hughes L., Cooke R. (2003). Motor, cognitive, and behavioural disorders in children born very preterm. Dev. Med. Child Neurol..

[b0090] Garfinkle J., Guo T., Synnes A. (2020). Location and size of preterm cerebellar hemorrhage and childhood development. Ann. Neurol..

[b0095] George J.M., Boyd R.N., Colditz P.B. (2015). PPREMO: a prospective cohort study of preterm infant brain structure and function to predict neurodevelopmental outcome. BMC Pediatr..

[b0100] George J.M., Fiori S., Fripp J. (2017). Validation of an MRI brain injury and growth scoring system in very preterm infants scanned at 29- to 35-week postmenstrual age. Am. J. Neuroradiol..

[b0105] George J.M., Fiori S., Fripp J. (2018). Relationship between very early brain structure and neuromotor, neurological and neurobehavioral function in infants born< 31 weeks gestational age. Early Hum. Dev..

[b0110] George J.M., Pannek K., Rose S.E. (2018). Diagnostic accuracy of early magnetic resonance imaging to determine motor outcomes in infants born preterm: a systematic review and meta‐analysis. Dev. Med. Child Neurol..

[b0115] George J.M., Pagnozzi A.M., Bora S. (2020). Prediction of childhood brain outcomes in infants born preterm using neonatal MRI and concurrent clinical biomarkers (PREBO-6): study protocol for a prospective cohort study. BMJ Open.

[b0120] George J.M., Colditz P.B., Chatfield M.D. (2021). Early clinical and MRI biomarkers of cognitive and motor outcomes in very preterm born infants. Pediatr. Res..

[b0125] Henderson S.E., Sugden D., Barnett A.L. (2007).

[b0130] Inder T.E., De Vries L.S., Ferriero D.M. (2021). Neuroimaging of the preterm brain: review and recommendations. J. Pediatrics..

[b0135] Inder T.E., Volpe J.J., Anderson P.J. (2023). Defining the neurologic consequences of preterm birth. N. Engl. J. Med..

[b0140] Janvier A., Farlow B., Baardsnes J. (2016). Measuring and Communicating Meaningful Outcomes in Neonatology: a Family Perspective. Seminars in Perinatology.

[b0145] Kidokoro H., Neil J.J., Inder T.E. (2013). New MR imaging assessment tool to define brain abnormalities in very preterm infants at term. Am. J. Neuroradiol..

[b0150] Kidokoro H., Anderson P.J., Doyle L.W. (2014). Brain injury and altered brain growth in preterm infants: predictors and prognosis. Pediatrics.

[b0155] McGowan E.C., Vohr B.R. (2019). Neurodevelopmental follow-up of preterm infants: what is new?. Pediatr. Clin..

[b0160] Ohuma E.O., Moller A.-B., Bradley E. (2023). National, regional, and global estimates of preterm birth in 2020, with trends from 2010: a systematic analysis. Lancet.

[b0165] Pagnozzi A.M., van Eijk L., Pannek K. (2023). Early brain morphometrics from neonatal MRI predict motor and cognitive outcomes at 2-years corrected age in very preterm infants. Neuroimage.

[b0170] Pannek K., George J.M., Cespedes M. (2024). Semiquantitative MRI scores for preterm infants are not consistent between protocols.

[b0175] Sie L., Hart A., Van Hof J. (2005). Predictive value of neonatal MRI with respect to late MRI findings and clinical outcome. A study in infants with periventricular densities on neonatal ultrasound. Neuropediatrics.

[b0180] Smyser C.D., Kidokoro H., Inder T.E. (2012). Magnetic resonance imaging of the brain at term equivalent age in extremely premature neonates: to scan or not to scan?. J. Paediatr. Child Health.

[b0185] Spittle A.J., Dewey D., Nguyen T.-N.-N. (2021). Rates of developmental coordination disorder in children born very preterm. J. Pediatr..

[b0190] Stoodley C.J., Limperopoulos C. (2016). Structure–function relationships in the developing cerebellum: evidence from early-life cerebellar injury and neurodevelopmental disorders. Semin. Fetal Neonatal Med..

[b0195] van’t Hooft J., van der Lee J.H., Opmeer B.C. (2015). Predicting developmental outcomes in premature infants by term equivalent MRI: systematic review and meta-analysis. Syst. Rev..

