# Supplementary material for: Diagnostic accuracy of neonatal structural MRI scores to predict 6-year motor outcomes of children born very preterm
Source: Neuroimage Clin. 2024 Dec 14;45:103725. doi: 10.1016/j.nicl.2024.103725 (PMC11721883; doi:10.1016/j.nicl.2024.103725)
Supplement: Supplementary Data 1 [file mmc1.docx]

**SUPPLEMENT: eMethods**

**MRI Acquisition Sequences**

The infants in the PPREMO study underwent axial T1 TSE (TR/TE 1490/90 ms; flip angle 150°; FOV 200 x 160 mm; matrix 256 x 180; slice thickness 2 mm), coronal, sagittal and axial T2-weighted HASTE (TR/TE 2000/90 ms; flip angle 150°; FOV 200 x 160 mm; matrix 320 x 256; slice thickness 4mm), and axial multi-echo T2 TSE images (TR/TE1/TE2/TE3 10,580/27/122/189 ms; flip angle 150°; FOV 144 x 180 mm; matrix 204 x 256; slice thickness 2 mm). Infants participating in the PREBO study underwent axial axial T1 3D FLASH (TR/TE 20/4.92 ms; flip angle 25; FOV 180 x 154.26 mm; matrix 224 x 192; 144 slices; slice thickness 0.8mm) and, coronal, axial, and sagittal T2-weighted HASTE (TR/TE 2280/117 ms; flip angle 120°; FOV 162.6[sagittal]/137.6[coronal,axial] x 200 mm; matrix 208[sagittal]/176[coronal,axial] x 256; slice thickness 1.8mm) (Pannek et al., 2024). Both T1 and T2 scans were used for MRI scoring as previously described (George et al., 2021).

**Participant Flow**

*N=*194 PPREMO+PREBO cohorts

*N=*194 Early MRI performed

*N=*173 Repeat MRI at term performed

*N=*123 Returned for 6-year follow-up

*N=*123 Final cohort

**SUPPLEMENT: eTables**

**eTable1.** Brain MRI categorical scores displayed by level of severity for Early and term equivalent age MRI

| MRI Scores | None | Mild | Moderate | Severe |
| --- | --- | --- | --- | --- |
| Early MRI (n=123) |  |  |  |  |
| White matter | 18 (15%) | 68 (55%) | 17 (14%) | 20 (16%) |
| Cortical grey matter | 79 (65%) | 30 (24%) | 14 (11%) | 0 (0%) |
| Deep gray matter | 68 (55%) | 35 (55%) | 13 (11%) | 7 (6%) |
| Cerebellum | 81 (66%) | 25 (20%) | 11 (9%) | 6 (5%) |
| GBAS | 34 (28%) | 57 (56%) | 21 (17%) | 11 (9%) |
| TEA MRI (n=114) |  |  |  |  |
| White matter | 71 (62%) | 22 (19%) | 9 (8%) | 12 (11%) |
| Cortical grey matter | 78 (68%) | 12 (11%) | 20 (18%) | 4 (3%) |
| Deep gray matter | 72 (63%) | 25 (22%) | 14 (12%) | 3 (3%) |
| Cerebellum | 80 (70%) | 21 (18%) | 9 (8%) | 4 (4%) |
| GBAS | 61 (54%) | 35 (31%) | 7 (6%) | 11 (9%) |
| Key: Data presented as number (%); GBAS, global brain abnormality score | | | | |

**eTable 2.** Adjusted and unadjusted regression analysis of association between MRI and MABC-2 total score at 6 years corrected age

|  | Unadjusted | | | Adjusted^α^ | | |
| --- | --- | --- | --- | --- | --- | --- |
| Parameter | **Coefficient** | **95% CI** | **P-value** | **Coefficient** | **95% CI** | ***P*-value** |
| Early MRI (*n*=116) |  |  |  |  |  |  |
| WM | -2.79 | [-4.52, -1.04] | <0.01* | -2.75 | [-4.48, -1.02] | <0.01* |
| CGM | -2.91 | [-8.26, 2.44] | 0.28 | -2.10 | [-7.22, 3.01] | 0.42 |
| DGM | -4.99 | [-8.16, -1.82] | <0.01* | -4.95 | [-8.08, -1.81] | <0.01* |
| Cerebellum | -6.20 | [-9.78, -2.62] | <0.01* | -6.67 | [-10.42, -2.92] | <0.01* |
| GBAS | -1.82 | [-2.75, -0.88] | <0.001* | -1.92 | [-2.88, -0.96] | <0.001* |
| TEA MRI (*n*=108) |  |  |  |  |  |  |
| WM | -2.09 | [-3.72, -0.46] | 0.01* | -1.75 | [-3.37, -0.13] | 0.04* |
| CGM | -5.98 | [-10.34, -1.61] | 0.01* | -5.53 | [-9.66, -1.41] | 0.01* |
| DGM | -4.90 | [-8.96, -0.84] | 0.02* | -3.95 | [-7.89, -0.002] | 0.05 |
| Cerebellum | -7.10 | [-10.9, -3.22] | <0.001* | -6.79 | [-10.70, -2.88] | <0.01* |
| GBAS | -1.82 | [-2.79, -0.86] | <0.001* | -1.67 | [-2.64, -0.69] | <0.01* |
| Key: TEA, term equivalent age; WM, white matter; CGM; cortical grey matter; DGM, deep grey matter; *statistically significant; ^α^adjusted for gestational age, sex and corrected age at motor assessment | | | | | | |

**eTable3.** Sensitivity and specificity of Early and TEA MRI scores to predict MABC-2 ≤15^th^ percentile score at 6 years corrected age. (results from 2x2 tables)

| Variable | Sensitivity  [95% CI] | Specificity  [95% CI] | Positive likelihood ratio [95% CI] | Negative likelihood ratio [95% CI] | Correctly classified (%) |
| --- | --- | --- | --- | --- | --- |
| Early MRI, *n*=116 | | | |  |  |
| WM | 35 [26, 43] | 76 [68, 84] | 1.45 [0.82, 2.58] | 0.86 [0.67, 1.10] | 59 |
| CGM | 12 [6, 18] | 90 [84, 95] | 1.17 [0.42, 24.77] | 0.98 [0.86, 1.12] | 57 |
| DGM | 27 [19, 35] | 94 [90, 98] | 4.44 [1.54, 12.81] | 0.78 [0.65, 0.93] | 66 |
| Cerebellum | 20 [13, 28] | 90 [84, 95] | 1.95 [0.80, 4.77] | 0.89 [0.75, 1.05] | 60 |
| GBAS | 35 [26, 43] | 84 [77, 90] | 2.11 [1.09, 4.10] | 0.78 [0.62, 0.98] | 63 |
| TEA MRI, *n*=108 | | | |  |  |
| WM | 29 [20, 37] | 92 [87, 97] | 3.64 [1.40, 9.49] | 0.77 [0.63, 0.94] | 66 |
| CGM | 24 [16, 33] | 86 [79, 92] | 1.71 [0.77, 3.78] | 0.88 [0.73, 1.07] | 60 |
| DGM | 18 [11, 25] | 89 [83, 95] | 1.60 [0.63, 4.09] | 0.93 [0.79, 1.09] | 59 |
| Cerebellum | 16 [9, 22] | 92 [87, 97] | 1.96 [0.66, 5.78] | 0.92 [0.79, 1.06] | 60 |
| GBAS | 27 [18, 35] | 95 [91, 99] | 5.60 [1.68, 18.70] | 0.77 [0.64, 0.93] | 67 |
| Key: Presented data in % and % [95% CI]; WM, white matter; CGM, cortical grey matter; DGM, deep grey matter; GBAS, global brain abnormality score | | | | | |

**eTable 4.** Sensitivity and specificity of Early and TEA MRI categorical scores to predict MABC-2 at cut-off scores of ≤5^th^ and ≤15^th^ percentiles at 6 years corrected age using ROC curves

| ≤5^th^ percentile cut-off score | | | | | | | ≤15^th^ percentile cut-off score | | | | | | | | |
| --- | --- | --- | --- | --- | --- | --- | --- | --- | --- | --- | --- | --- | --- | --- | --- |
| Variable | **MRI Cut-off point** | **Sensitivity (%)** | **Specificity (%)** | **Correctly classified with ROC (%)** | **Correctly Classified as per 2x2 table (%)** | **ROC area [95% CI]** | **MRI Cut-off point^α^** | **Sensitivity (%)** | | **Specificity (%)** | **Correctly classified with ROC (%)** | | **Correctly Classified as per 2x2 table (%)** | | **ROC area [95% CI]** |
| Early MRI (*n*=116) | | | | | | | | | | | | | | | |
| WM | ≥Sev | 26 | 92 | 70 | 64 | 0.6 [0.5­–0.7] | ≥Sev | | 23 | 93 | 63 | | 59 | | 0.6 [0.5–0.7] |
| CGM | ≥Mild | 38 | 66 | 57 | 64 | 0.5 [0.4–0.6] | ≥Mod | | 12 | 90 | 57 | | 57 | | 0.5 [0.4–0.6] |
| DGM | ≥Mod | 26 | 91 | 69 | 69 | 0.5 [0.4–0.7] | ≥Mod | | 27 | 94 | 66 | | 66 | | 0.6 [0.5–0.7] |
| Cerebellum | ≥Mod | 23 | 90 | 67 | 67 | 0.6 [0.5­–0.7] | ≥Mild | | 43 | 72 | 60 | | 60 | | 0.6 [0.5–0.7] |
| GBAS | ≥Mod | 36 | 82 | 66 | 66 | 0.6 [0.5–0.7] | ≥Mod | | 35 | 84 | 63 | | 63 | | 0.6 [0.5–0.7] |
| TEA MRI (*n=*108) | | | | | | | | | | | |  | |  | |
| WM | ≥Mod | 31 | 90 | 70 | 70 | 0.6 [0.5–0.7] | ≥Mod | | 29 | 92 | 66 | | 66 | | 0.6 [0.5–0.7] |
| CGM | ≥Mild | 39 | 78 | 65 | 65 | 0.6 [0.5–0.7] | ≥Mild | | 36 | 78 | 60 | | 60 | | 0.6 [0.5–0.7] |
| DGM | ≥Mild | 44 | 67 | 59 | 64 | 0.6 [0.5–0.7] | ≥Mild | | 42 | 67 | 56 | | 59 | | 0.6 [0.5–0.7] |
| Cerebellum | ≥Mod | 19 | 93 | 69 | 69 | 0.6 [0.5–0.7] | ≥ Mild | | 36 | 78 | 60 | | 60 | | 0.6 [0.5–0.7] |
| GBAS | ≥Mod | 28 | 93 | 71 | 71 | 0.5 [0.4–0.7] | ≥Mod | | 27 | 95 | 67 | | 67 | | 0.6 [0.5–0.7] |
| Key: Presented data in %; WM, white matter; CGM, cortical grey matter; DGM, deep grey matter; GBAS, global brain abnormality score; Sev, severe; Mod, moderate | | | | | | | | | | | | | | | |

**eTable 5.** Sensitivity and specificity of Early and TEA MRI continuous scores to predict MABC-2 at cut-off scores of ≤5^th^ and ≤15^th^ percentiles at 6 years corrected age using ROC curves

| ≤5^th^ percentile cut-off score | | | | | | | ≤15^th^ percentile cut-off score | | | | | | | | |
| --- | --- | --- | --- | --- | --- | --- | --- | --- | --- | --- | --- | --- | --- | --- | --- |
| Variable | **MRI Cut-off point score** | **Sensitivity (%)** | **Specificity (%)** | **Correctly classified with ROC (%)** | **Correctly Classified as per 2x2 table (%)** | **ROC area [95% CI]** | **MRI Cut-off point** | **Sensitivity (%)** | | **Specificity (%)** | **Correctly classified with ROC (%)** | | **Correctly Classified as per 2x2 table (%)** | | **ROC area [95% CI]** |
| Early MRI (*n*=116) | | | | | | | | | | | | | | | |
| WM | ≥ 7 | 26 | 92 | 70 | 64 | 0.6 [0.5­–0.7] | ≥6 | | 29 | 88 | 63 | | 59 | | 0.6 [0.5–0.7] |
| CGM | ≥1 | 38 | 66 | 57 | 64 | 0.5 [0.4–0.6] | ≥1 | | 37 | 66 | 53 | | 57 | | 0.5 [0.4–0.6] |
| DGM | ≥2 | 26 | 91 | 69 | 69 | 0.5 [0.4–0.7] | ≥2 | | 27 | 94 | 66 | | 66 | | 0.6 [0.5–0.7] |
| Cerebellum | ≥2 | 23 | 90 | 67 | 67 | 0.6 [0.5–0.7] | ≥1 | | 43 | 72 | 60 | | 60 | | 0.6 [0.5–0.7] |
| GBAS | ≥9 | 33 | 88 | 70 | 66 | 0.6 [0.5–0.7] | ≥9 | | 33 | 91 | 66 | | 63 | | 0.6 [0.5–0.7] |
| TEA MRI (*n=*108) | | | | | | | | | | | |  | |  | |
| WM | ≥5 | 31 | 90 | 70 | 70 | 0.5 [0.4–0.6] | ≥5 | | 29 | 92 | 66 | | 66 | | 0.5 [0.4–0.7] |
| CGM | ≥1 | 39 | 78 | 65 | 65 | 0.6 [0.5–0.7] | ≥1 | | 36 | 78 | 60 | | 60 | | 0.6 [0.5–0.7] |
| DGM | ≥1 | 44 | 67 | 59 | 64 | 0.6 [0.5–0.7] | ≥1 | | 42 | 67 | 56 | | 59 | | 0.6 [0.5–0.7] |
| Cerebellum | ≥2 | 19 | 93 | 69 | 69 | 0.6 [0.5–0.7] | ≥1 | | 36 | 78 | 60 | | 60 | | 0.6 [0.5–0.7] |
| GBAS | ≥6 | 36 | 90 | 72 | 71 | 0.5 [0.4–0.7] | ≥6 | | 33 | 92 | 68 | | 67 | | 0.6 [0.5–0.7] |
| Key: Presented data in %; WM, white matter; CGM, cortical grey matter; DGM, deep grey matter; GBAS, global brain abnormality score | | | | | | | | | | | | | | | |

**eTable 6.** Sensitivity and specificity of Early and TEA MRI categorical and continuous scores to predict CP at 6 years corrected age using ROC curves

| CP and MRI categorical score | | | | | | | CP and MRI continuous scores | | | | | | | | |
| --- | --- | --- | --- | --- | --- | --- | --- | --- | --- | --- | --- | --- | --- | --- | --- |
| Variable | **MRI Cut-off point** | **Sensitivity (%)** | **Specificity (%)** | **Correctly classified with ROC (%)** | **Correctly Classified as per 2x2 table (%)** | **ROC area [95% CI]** | **MRI Cut-off point** | **Sensitivity (%)** | | **Specificity (%)** | **Correctly classified with ROC (%)** | | **Correctly Classified as per 2x2 table (%)** | | **ROC area [95% CI]** |
| Early MRI (*n*=116) | | | | | | | | | | | | | | | |
| WM | ≥ Sev | 78 | 89 | 88 | 74 | 0.8 [0.6–1.0] | ≥7 | | 78 | 89 | 88 | | 74 | | 0.8 [0.6–1.0] |
| CGM | ≥Mild | 56 | 66 | 65 | 83 | 0.6 [0.4–0.8] | ≥1 | | 56 | 66 | 65 | | 83 | | 0.6 [0.4–0.8] |
| DGM | ≥Mod | 78 | 89 | 88 | 88 | 0.8 [0.6–1.0] | ≥2 | | 78 | 89 | 88 | | 88 | | 0.8 [0.6–1.0] |
| Cerebellum | ≥Mod | 33 | 88 | 84 | 84 | 0.6 [0.4­–0.8] | ≥2 | | 33 | 88 | 84 | | 84 | | 0.6 [0.4­–0.8] |
| GBAS | ≥Mod | 78 | 78 | 78 | 78 | 0.8 [0.6–1.0] | ≥11 | | 78 | 93 | 92 | | 78 | | 0.8 [0.6–1.0] |
| TEA MRI (*n=*108) | | | | | | | | | | | |  | |  | |
| WM | ≥Sev | 75 | 94 | 93 | 85 | 0.8 [0.8–1.0] | ≥8 | | 75 | 99 | 97 | | 85 | | 0.8 [0.5–1.0] |
| CGM | ≥Mild | 75 | 72 | 72 | 77 | 0.7 [0.5–0.9] | ≥1 | | 75 | 72 | 72 | | 77 | | 0.7 [0.5–0.9] |
| DGM | ≥Mild | 63 | 65 | 65 | 82 | 0.6 [0.4–0.8] | ≥1 | | 63 | 65 | 65 | | 82 | | 0.6 [0.4–0.8] |
| Cerebellum | ≥Mod | 50 | 92 | 89 | 89 | 0.7 [0.5–0.9] | ≥2 | | 50 | 92 | 89 | | 89 | | 0.7 [0.5–0.9] |
| GBAS | ≥Mod | 75 | 89 | 88 | 88 | 0.8 [0.6–1.0] | ≥10 | | 75 | 93 | 92 | | 88 | | 0.8 [0.5–1.0] |
| Key: Presented data in %; WM, white matter; CGM, cortical grey matter; DGM, deep grey matter; GBAS, global brain abnormality score | | | | | | | | | | | | | | | |

**SUPPLEMENT: Figures**

| **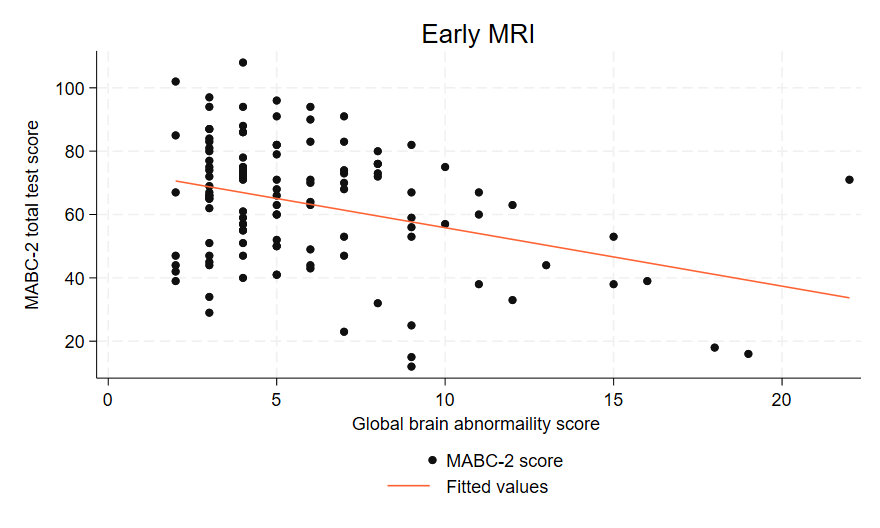** | **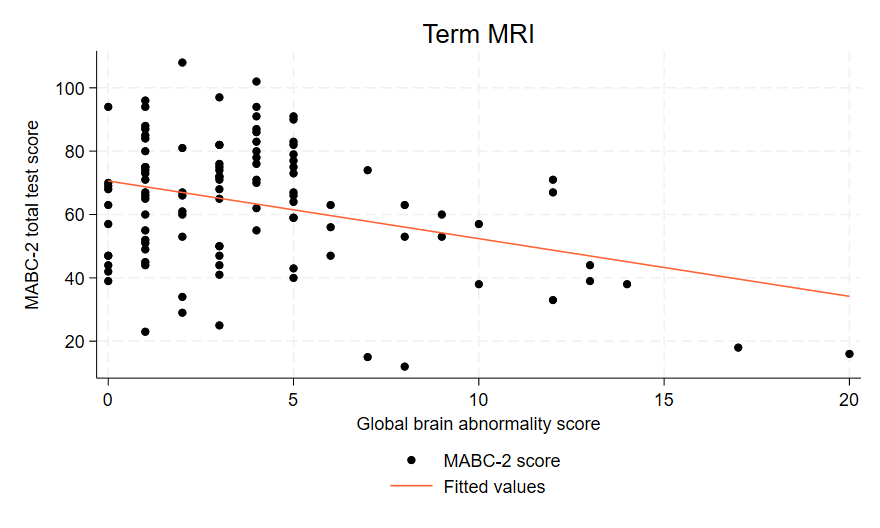** |
| --- | --- |
| **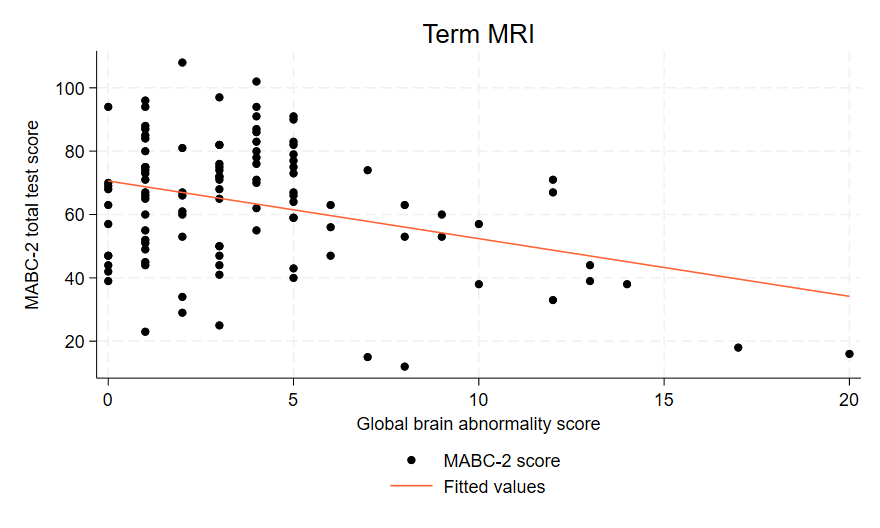** | |
| **eFigure 1. Scatter plot demonstration relationship between MRI and MABC-2 score**  **Description:** Relationship between Early (left) and Term (right) MRI global brain abnormaility score with Movement Assessment Battery for Children second edition (MABC-2) total test score | |

| 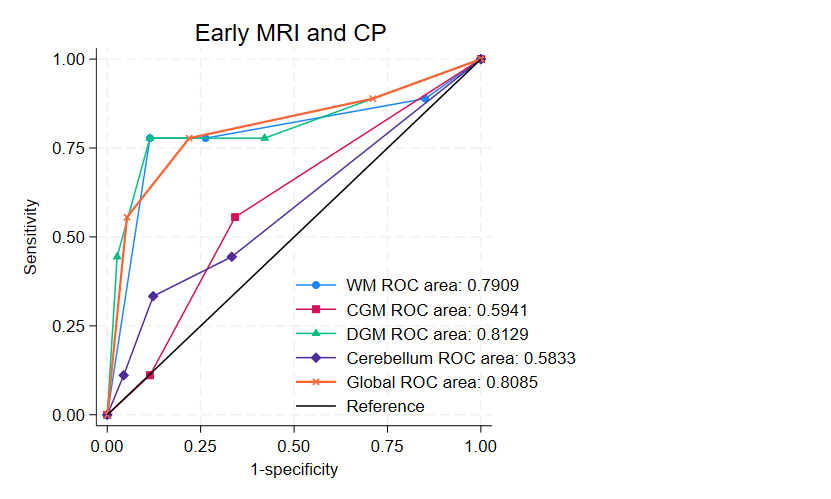 | 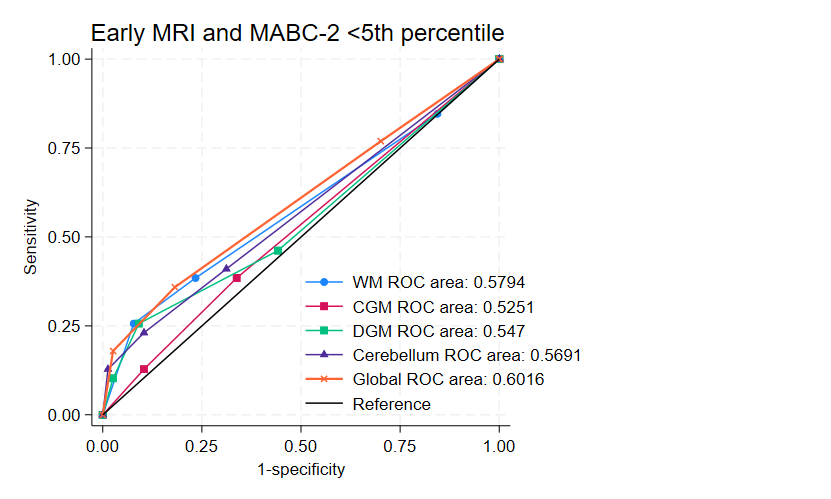 | 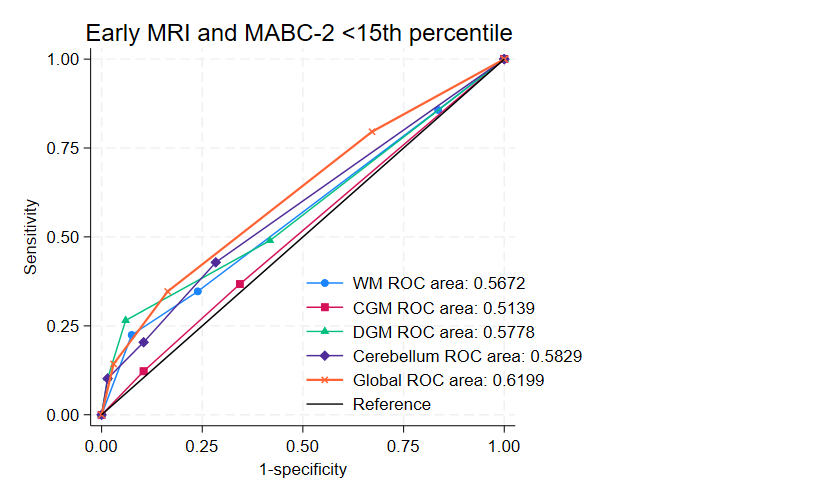 |
| --- | --- | --- |
| 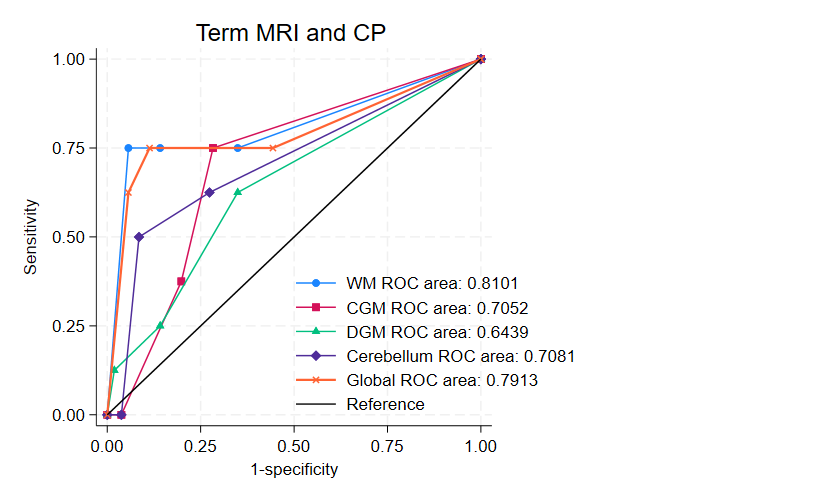 | 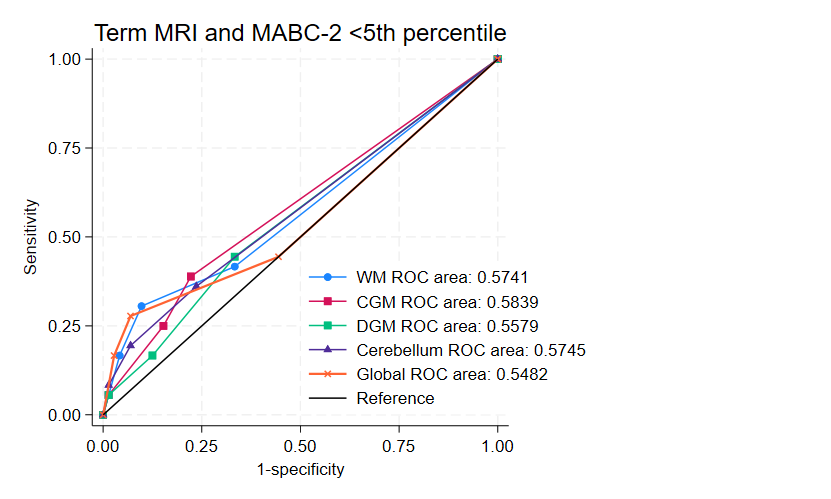 | 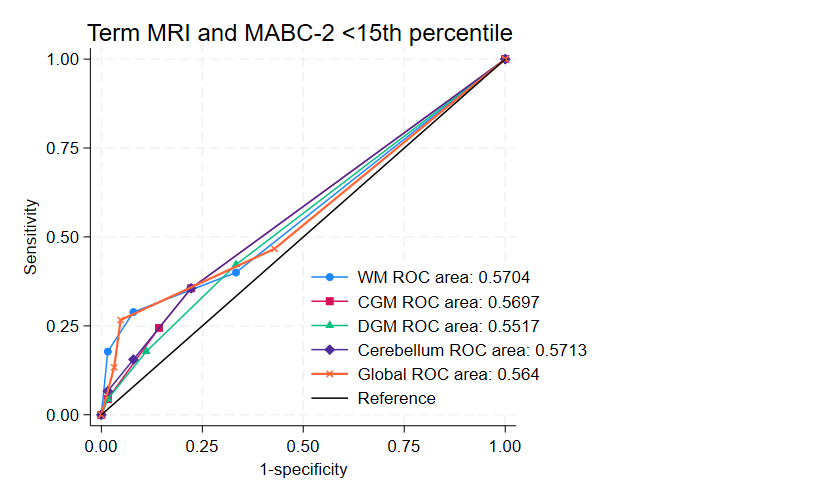 |
| **eFigure 2. Receiver operating characteristic curves for categorical MRI scores to predict motor outcomes**  **Description:** ROC curves for Early (first row) and Term MRI (Second row), white matter (WM), cortical grey matter (CGM), deep grey matter (DGM), cerebellum and global brain abnormality categorical scores (none, mild, moderate, severe) to predict adverse motor outcomes on CP (first column), Movement Assessment Battery for Children second edition (MABC-2) ≤5^th^ percentile score (second column) and ≤15^th^ percentile score (third column) at 6.1 years CA. | | |

| 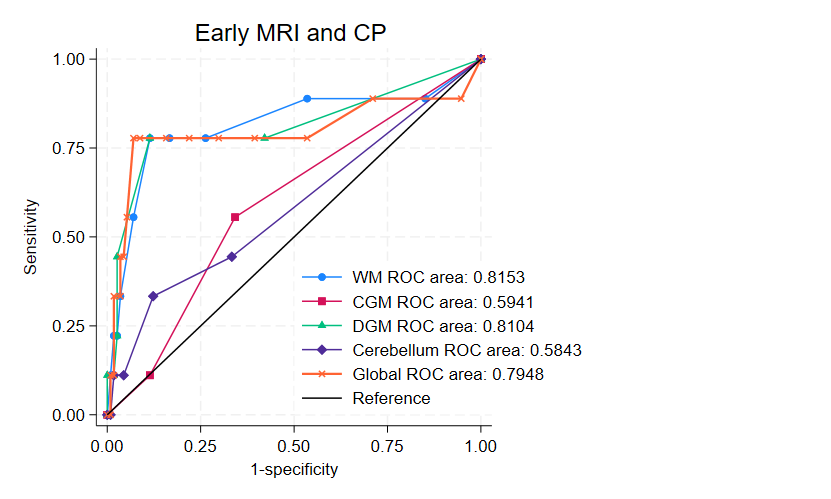 | 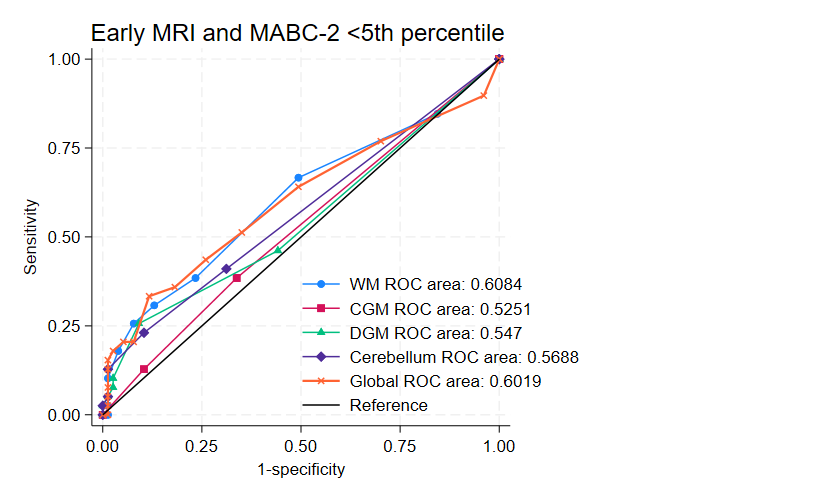 | 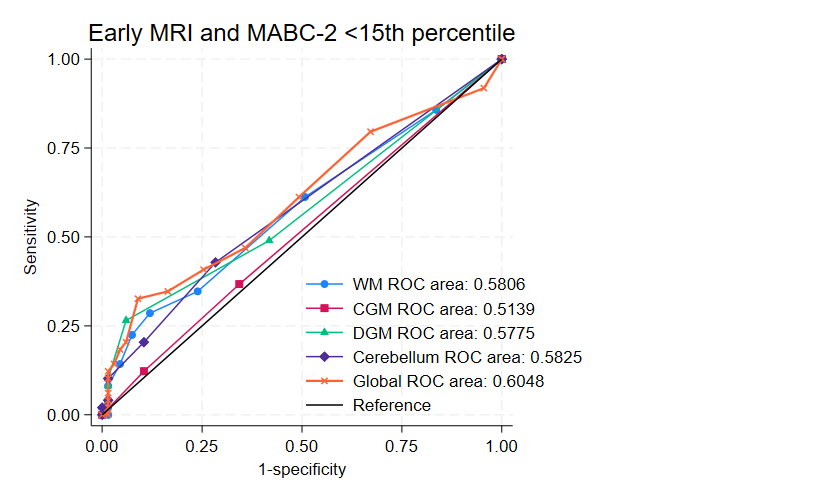 |
| --- | --- | --- |
| 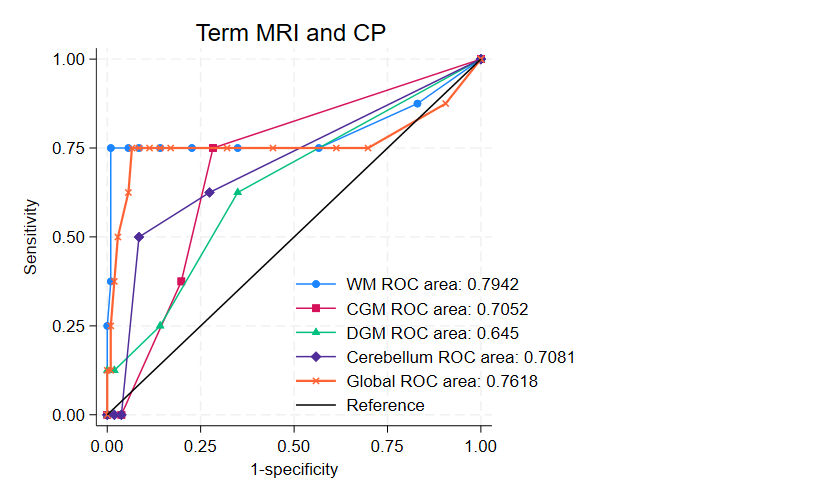 | 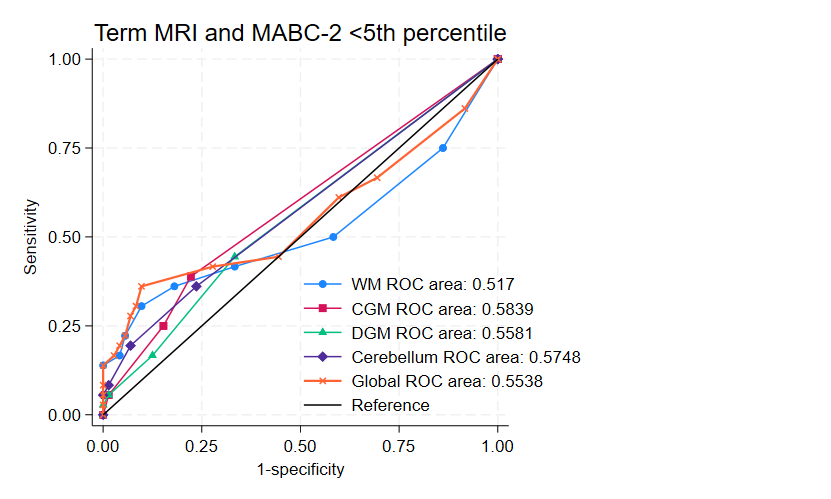 | 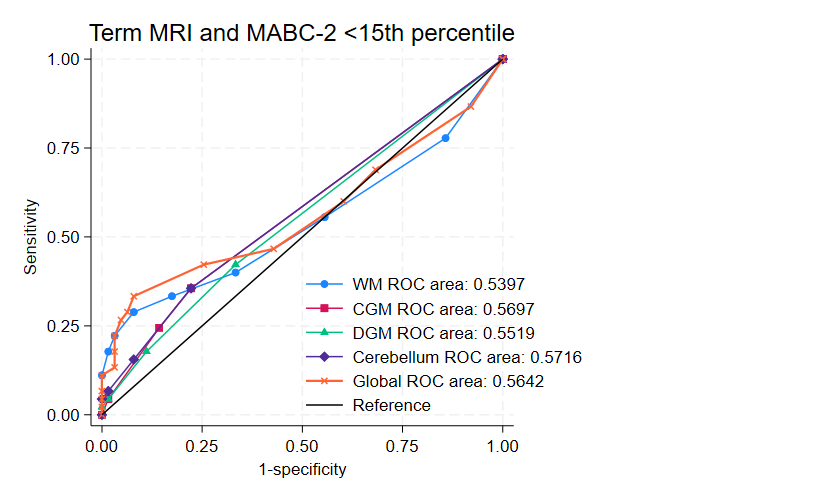 |
| **eFigure 3. Receiver operating characteristic curves for continuous MRI scores to predict motor outcomes**  **Description:** ROC curves for Early (first row) and Term MRI (Second row), white matter (WM), cortical grey matter (CGM), deep grey matter (DGM), cerebellum and global brain abnormality score (continuous) to predict adverse motor outcomes on CP (first column), Movement Assessment Battery for Children second edition (MABC-2) ≤5^th^ percentile score (second column) and ≤15^th^ percentile score (third column) at 6.1 years CA. | | |

**Reference List**

1. George JM, Colditz PB, Chatfield MD, et al. Early clinical and MRI biomarkers of cognitive and motor outcomes in very preterm born infants. Pediatric Research. 2021;90(6):1243-50.

2. Pannek K, George JM, Cespedes M, et al. Semiquantitative MRI scores for preterm infants are not consistent between protocols. 12th AusACPDM Biennial Conference Cairns, Australia DMCN 2024. p. 5–99.
